# Barriers to Implementing Shared Decision-Making in Postgraduate Medical Education: The Role of Disease-Centered Beliefs

**DOI:** 10.5334/pme.1465

**Published:** 2025-07-25

**Authors:** Laura Alexandra van der Woude, Gera A. Welker, Paul L. P. Brand, Suzanne Festen

**Affiliations:** 1 University Medical Center Groningen (UMCG), Hanzeplein 1, 9713 GZ Groningen, The Netherlands; 2 Isala Academy, Department of Medical Education and Faculty Development, Isala, Dokter van Heesweg 2, 8025 AB Zwolle, The Netherlands; 3 Wenckebach Institute for Medical Education, University of Groningen and University Medical Center Groningen, Groningen, The Netherlands; 4 University of Groningen, University Center for Geriatric Medicine, University Medical Center Groningen (UMCG), Hanzeplein 1, 9713 GZ Groningen, The Netherlands

## Abstract

**Introduction:**

Despite the well-documented benefits of shared decision-making (SDM), its implementation in practice remains limited. Efforts to promote SDM often fail to produce lasting behavioral change among physicians. Underlying conscious or unconscious beliefs may shape their decision-making processes, influencing the extent to which SDM is applied. This study aimed to explore the perceptions, beliefs and behaviors of Dutch residents and medical specialists regarding SDM and to identify potential barriers to its integration into postgraduate medical education.

**Methods:**

A mixed-method study was conducted, involving a survey (comprising control preference scale (CPS) and iSHARE) and focus group interviews among residents and medical specialists from seven Dutch teaching hospitals.

**Results:**

SDM was supported by 93% (292/315) of survey respondents, with 89% (280/315) agreeing that it should be an integral part of postgraduate medical education. Seven residents (6%) and 33 medical specialists (18%) indicated they had followed an SDM training. Thematic analysis of the focus group interviews identified four disease-centered beliefs that influenced clinical thinking and decision-making among both residents and medical specialists. This disease-centeredness emerged as the primary barrier to the successful implementation of SDM.

**Discussion:**

While SDM is widely endorsed, its practical implementation is constrained by disease-centered thinking. Achieving sustainable integration of SDM in postgraduate medical education requires a fundamental paradigm shift, in which residents and medical specialists become aware of their disease-centered beliefs and instead learn to think and act in a more person-centered manner.

## 1. Introduction

Shared decision making (SDM) is the process in which healthcare professionals and patients decide together which medical decision best suits the patient’s medical situation, context, personal preferences and goals [1,2]. A well executed SDM process consists of four key elements: introducing choice, describing options with their pros and cons, exploring patient preferences and supporting the patient with the decision-making [3]. SDM goes hand in hand with person-centered care, in which patients’ values and preferences guide all aspects of their care. SDM can provide the necessary framework for a transition to person-centered care [4], while a person-centered attitude is needed for the optimal implementation of SDM [5].

When asked, most patients state a preference to be actively involved in making decisions regarding their health [6]. The main goal of SDM is to promote and respect patient autonomy [3]. SDM is associated with higher patient satisfaction, less decisional regret, more appropriate treatments, better adherence to treatment and lower healthcare costs [7,8,9,10,11]. As a result, SDM has been adopted as the preferred decision-making model by governments, patients’ and healthcare professionals’ organizations [12,13].

Although most doctors say they prefer to make shared decisions, implementation in practice and medical education appears to be difficult [14,15,16]. Previously mentioned barriers to SDM include lack of time, emergency setting, patients’ limited health literacy and doctors’ lack of SDM skills [15,17,18,19]. Moreover, recent research shows that residents have a strong belief that they are responsible for choosing the best treatment for their patients, which is likely to be a barrier for the application of SDM [17].

This finding raises the question if other conscious or unconscious beliefs are at play in doctors’ decision-making, if these beliefs occur among both residents and medical specialists, and how these affect the clinical decision-making practice. This study aimed to investigate the attitudes, beliefs and behavior regarding SDM among Dutch residents and medical specialists (i.e., the residents’ supervisors). The study was designed to provide a basis for an evidence-based medical education intervention to improve SDM and a person-centered approach.

## 2. Methods

### Study design

A mixed-method study, comprising a survey and focus group interviews among residents and medical specialists from seven teaching hospitals in the Netherlands’ northeastern educational region.

### Recruitment

The northeastern educational region comprises the University Medical Center of Groningen and six affiliated general teaching hospitals in four provinces (Groningen, Friesland, Drenthe and Overijssel). An information letter with a link to a digital, anonymous survey was emailed by each hospital’s education committee to all residents and (associate) program directors. The survey remained open for three months (June– September 2022). Email was chosen as the distribution method both for its ability to efficiently reach the entire region and because email surveys tend to yield higher response rates than paper surveys. To further boost participation, a reminder email was sent during the survey period [20]. After completion of survey data acquisition, an information letter regarding the focus groups was sent to the same group of physicians. Participants who had expressed a special interest in the study when completing the survey were approached separately by email by one of the authors. Purposive sampling was used to include participants from different disciplines and hospitals. Focus groups were set up in a hybrid format to facilitate both live and online (Microsoft Teams) participation. All participants provided written informed consent.

### Data collection

The survey consisting of two validated questionnaires (Control Preference Scale and iSHARE [21,22]) and free text questions was used to explore doctors’ current self-reported skills and attitudes regarding SDM (Appendix A). The focus group interviews were used to clarify views expressed in the survey, to obtain more detailed information on doctors’ attitudes about the SDM process, and to identify possible barriers for implementing SDM in postgraduate medical education. Each focus group interview lasted approximately 1.5 hours and followed a semistructured interview guide of four main research questions complementing the findings of the survey and previous research [17] (Appendix B). Focus groups were led by a qualitative researcher with considerable expertise in conducting focus group interviews (GW). The first author (LW) attended the focus group interviews, took field notes and asked additional questions when needed. After each interview, the interviewers’ field notes and impressions were shared with the other authors. These discussions were used to identify emerging themes and modify the interview guide as needed. The focus group interviews were videotaped and transcribed verbatim. Participants were pseudonymized in transcripts. Their age, gender, residency year, work experience and type of hospital were recorded as descriptive characteristics. The Consolidated criteria for reporting qualitative research (COREQ) [23] were followed during the entire process (Appendix C). The University Medical Center Groningen medical ethics committee approved the study (file number: 202200284). Participants received a €15 gift card as compensation for attending the focus group interview.

### Data analysis

Descriptive statistics were used to analyse the survey results. A thematic analysis was chosen to systematically analyse the focus group interviews [24]. Two approaches were used: a deductive approach, using data of preconceived themes based on existing knowledge, and an inductive approach, allowing the data of the interviews to determine the themes. This method seemed most suitable as this allowed for the exploration of previously identified themes [17], supplemented with new themes developed from the data. The deductive themes that had emerged in previous research were: contextual factors (including patient factors, environmental factors), doctors’ medical and SDM skills and doctors’ beliefs [17]. These themes were used as a basis during the initial coding process. A comprehensive overview of the analytical process is shown in Appendix D. Initial coding was done independently by two authors (LW and SF), to promote reliability. Differences in coding were resolved by consensus. Themes and subthemes were discussed with the other members of the research team, modified and re-applied to the transcripts, until no more (sub)themes emerged. First, semantic codes were applied to represent what was literally said by participants. Latent codes were then used to identify possible underlying (unconscious) beliefs, assumptions and patterns that may play a role in decision-making. Latent codes and themes were identified by watching the focus group video recordings and paying special attention to intonation, group interaction (e.g. laughter, silences), issue absence or presence or so called ‘sign vehicles’ [25], which is a word or set of words that may carry extra meaning in the context of the interview. Vertical coding was used to identify similarities and differences between residents and medical specialists. All coding was done manually using word processor software (Microsoft Word).

### Research team and reflexivity

The first author (LW) is a fifth year hematology resident with previous experience in general surgery and intensive care. This broad clinical background gave her insight into participants’ work contexts. GW, a sociologist and implementation expert, coordinates the value-based healthcare program at the University Medical Center Groningen and brings a non-medical perspective. PB is professor in medical education and former pediatrician and researches doctor-patient communication. He is vice-chair of a Dutch Medical Specialists’ Federation committee which promotes the implementation of SDM in hospital practice. SF, an internist geriatrician, researches decision-making regarding treatment of vulnerable older patients and chairs a workgroup on implementing SDM in medical education. The team’s diverse backgrounds enabled a multifaceted analysis of focus group data. Conducting interviews with a non-medical specialist aimed to create an open, psychologically safe environment for participants, especially residents. The researchers aimed to approach data analysis with an open mind, acknowledging the complexity of clinical practice and recognizing, partly through their professional experience, that theory and practice do not always align. To gain a broader perspective on decision-making, they engaged in discussions with HZ, a clinical epidemiologist and professional coach for physicians, who brought a different perspective on decision-making. Any interpretative differences of codes and themes were resolved through discussion and consensus within the research team.

## 3. Results

### Survey

Of the 2058 physicians to whom the survey was sent, 331 completed it (response rate 16%). Sixteen respondents were excluded because they had no direct patient contact (e.g. pathologists). A total of 127 residents and 188 medical specialists were included in the results. An overview of all survey results is shown in Appendix A.

#### Current practice and attitudes regarding SDM

Nearly all respondents (292/315, 93%) expressed a preference for SDM when feasible. Self-reported application of SDM in clinical practice was noted by 46 residents (36%) and 116 medical specialists (62%). Almost half of the residents (60/127, 47%) and 27% of medical specialists (51/188) believed that most patients do not want to make shared decisions. Additionally, 67% of the respondents (208/315) felt that SDM takes more time than regular decision-making.

#### SDM in medical education

Most respondents (280/315, 89%) stated that SDM should be an integral part of postgraduate medical education. Seven residents (6%) and 33 medical specialists (18%) indicated they had followed an SDM training. Half of the respondents (164/315, 52%) reported they do not have several role models regarding SDM within their medical department. Fifty residents (40%, 50/127) said they do not get supervised on SDM.

### Focusgroup interviews

#### General characteristics

One focus group interview was conducted with 11 residents and two other focus group interviews were held with three and seven medical specialists, respectively. Participant characteristics are shown in Table 1.

**Table 1:** Characteristics of participants.


	RESIDENTS (n = 11)	MEDICAL SPECIALISTS (n = 10)

**Age (median in years)**	32 [26–35]	47 [33–65]

**Female (n %)**	9 (82%)	6 (60%)

**Residency year (n %)**		

*- year 1–2*	4 (36%)	

*- year 3–4*	6 (55%)	

*- year 5–6*	1 (9%)	

**Medical specialist, years in profession (n %)**		

*- 1–10 years*		2 (20%)

*- 11–20 years*		3 (30%)

*- 21–30 years*		2 (20%)

*- 31–40 years*		3 (30%)

**Specialty (n %)**		

*- Surgical*	4 (36%)	2 (20%)

*- Medical*	6 (55%)	6 (60%)

*- Primary care*	1 (9%)	2 (20%)

**Hospital (n %)**		

*- General hospital*	3 (27%)	3 (30%)

*- University hospital*	7 (64%)	5 (50%)

*- General practice*	1 (9%)	2 (20%)


#### Themes overview

**Figure 1:**
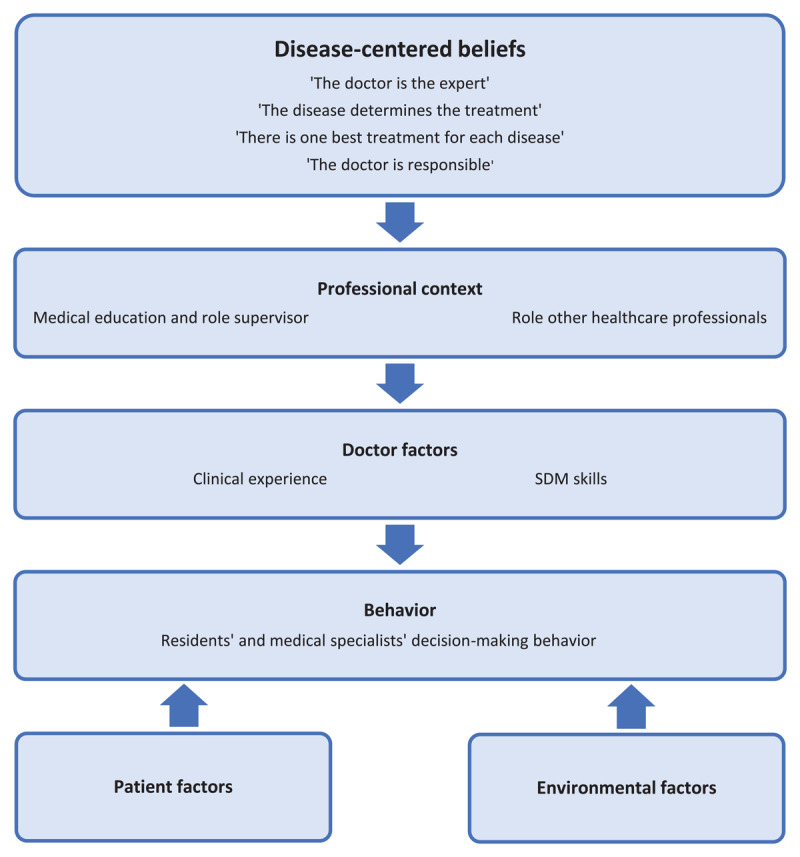
Interrelationship between themes.

We identified four main semantic themes concerning SDM: professional context, doctor factors, patient factors and environmental context (Figure 1). With latent coding, an overarching theme of disease-centeredness emerged, that appeared to underlie participants’ beliefs regarding SDM. The themes and associated key quotes are presented below. A table with additional quotes for each theme is shown in Appendix E.

### Semantic themes

#### Theme 1 – Professional context

##### Medical education and role supervisor

Residents indicated that their decision-making behavior was greatly influenced by the interaction with their supervisor. Residents felt that supervisors who only give one treatment option or make the decision for them, prevent them from applying SDM. They would rather receive information from their supervisor about the pros and cons of different treatment options and discuss these with the patient.

*“If you are going to consult with a supervisor to discuss the options, the supervisor usually makes the decision. Then you have to sell that to the patient and dress it up in such a way that it seems you made the decision together” – resident orthopedic surgery, 4^th^ year*

Participants felt that positive role models and attention to SDM in the clinical workplace or in medical education could facilitate SDM.

##### Role of other healthcare professionals

Participants expressed that patients sometimes discuss different issues with nurses than with doctors, which could give nurses an important role in the SDM process. The general practitioner (GP) was mentioned as an important factor in providing person-specific information, because the GP often knows the patient best.

*“Because there is a different hierarchy… they tell different things to the rheumatology nurse than to me, and that uhm sometimes really helps to make different choices” – rheumatologist, 26–30 years in profession*

#### Theme 2 – Doctor factors

##### Clinical experience

Both residents and medical specialists indicated that clinical experience was required for the adequate application of SDM. They mentioned that insufficient medical knowledge about the treatment options or prospects is a barrier for SDM.

*“It is difficult to find out what is important for the patient to take into account, when you have little experience with the treatment” – resident rheumatology, 4^th^ year*

Residents indicated that clinical experience also had an impact on their confidence in making decisions. Senior residents expressed that they felt more confident and were more likely to engage in SDM and to speak up to their supervisors if they did not agree with the plan.

*“I think you approach a conversation very differently if you have medical knowledge and experience. If you don’t have experience yet, you may have the impression that you just have to do what your supervisor says” – resident geriatrics, 2^nd^ year*

##### SDM skills

Regarding SDM skills, participants were not always aware of (the potential lack of) these skills and recognized that they could forget particular steps in the SDM process.

*“I think the pitfall for us is that we often outline the pros and cons and tell the patient to make the decision, but that is different from really asking ‘but what is important to you?’” – rheumatologist, 26–30 years in profession*

#### Theme 3 – Patient factors

Participants seemed to struggle with patients’ limited health literacy and considered this to be an important barrier for SDM. They reported that patients with a strong opinion or those who can express themselves well were more likely to participate in decision-making. Participants experienced that patients were sometimes too overwhelmed by emotions or too ill to engage in SDM. For example, if patients just received bad news, or in case of reduced level of consciousness due to severe illness.

*“Intelligence level is also such a complicated one, isn’t it? [agreement in the group]… So you quickly ask too much of the patient…you explain things, but very little is recalled” – psychiatrist, 11–15 years in profession*

#### Theme 4 – Environmental factors

According to participants, environmental factors like lack of time, setting (such as emergency room) and the availability of SDM tools (such as decision aids) influenced their application of SDM.

*“You can hardly explain and discuss everything with the patient in that amount of time, in such a way that it is actually understood and perhaps even discussed at home… you cannot actually ask a patient to make a decision in 5 or 10 minutes” – resident E.N.T., 1^st^ year*

### Main overarching latent theme: disease-centeredness

One main overarching theme, **disease-centeredness**, emerged from the data, shaping the medical thinking and decision-making behaviors of both residents and medical specialists. Disease-centeredness refers to a focus primarily on the characteristics of the disease itself, rather than on patient-specific factors such as individual goals, preferences or personal considerations [26]. This approach places greater value on objective, clinical disease parameters in decision-making, often to the exclusion or at the expense of subjective patient input [27]. Four key beliefs were identified that aligned with and reinforced this disease-centered approach.

#### Belief 1 – The doctor is the expert

Both residents and medical specialists viewed themselves as the primary experts in medical decision-making, while considering most patients as insufficiently capable of fully understanding the implications of their choices. This belief is grounded in the idea that expertise in disease lies with the physician, not the patient.

*“Before the patient can make an informed choice.. well, there is a reason why doctors have such a long education before they are able to make a choice like that.” – resident E.N.T., 1^st^ year*

Many participants expressed strong convictions regarding the best treatment for their patients and described efforts to steer patients toward their preferred treatment plan. This belief was perceived as a barrier to SDM.

*“I think in practice the story is presented in such a way that you push the patient to the direction you want to go, so yes, I think in practice the feeling of making decisions together is applied more than actually making decisions together” – resident orthopedic surgery, 4^th^ year*

#### Belief 2 – The disease determines the treatment

Participants placed primary emphasis on disease-specific characteristics when making treatment decisions. This approach was often described as a product of traditional medical education.

“*We are of course trained in a certain way, uhm, that we had to give a treatment proposal for a certain medical condition” – orthopedic surgeon, 11–15 years in profession*

Participants experienced that decisions were frequently made in advance, particularly in multidisciplinary team (MDT) meetings, where treatment plans were finalized before direct consultation with the patient. This was not generally viewed as problematic, and participants described it as a common practice.

*“To return to those MDT’s where the treatment plan has already been completely determined. I don’t think that is surprising. Very difficult cases are discussed and then you will go sit together with a group of specialists who, for example, all treat the same disease” – resident E.N.T., 4^th^ year*

When asked what additional patient-specific information was needed to guide treatment decisions, none of the participants mentioned the need to explore patient goals, preferences or values (*issue absence*).

#### Belief 3 – There is one best treatment for each disease

Participants strongly believed that each disease had a single best treatment, determined primarily by clinical guidelines and research evidence. This perspective contributed to the perception that decision-making often lacked real choice.

*“It depends if the treatment options are equal. If it is a choice like: ‘well we have option A and we have option B but we don’t necessarily know which is better, what is your preference?’. Ehm but if I know option A is better, well, am I going to give the patient a choice then?” – resident pediatrics, 4^th^ year*

Some participants expressed tension between respecting patient autonomy and their own medical judgment, particularly when a patient’s preference differed from the physician’s perception of the best treatment.

#### Belief 4 – The doctor is responsible

Participants expressed a strong sense of responsibility for their patients, encompassing the need to establish an accurate diagnosis, select the best treatment, and ensure the best possible outcomes. This belief reinforced the notion that the physician, as the expert, should make the final decision. Their perceived responsibility for treatment outcomes also influenced the extent to which participants involved patients in decisions, particularly when the stakes were high.

*“I think this [SDM] happens less with younger patients, especially in oncology. Yes you always have a choice of course, you always have to decide together of course, but ehm, doing nothing sometimes means that the patient dies” – resident general surgery, 5^th^ year*

Some participants indicated that their sense of responsibility made it difficult to discontinue treatments once initiated, even when doing so might align better with patient preferences.

*“When you have had a major revision because you have an infection and after that major surgery you get something minor, then it is a terrible shame to give up, also for the patient… In the beginning we frequently ask: do you want all this?, but at a certain moment, yeah, I think most of us do not have that conversation again. [confirmatory sounds]” – resident orthopedic surgery, 4^th^ year*

Residents also expressed skepticism about the reliability of patient-reported information, preferring to rely on other healthcare professionals for decision-making.

*“Then I finally called the GP and it was a completely different story than what the patient had told me. So sometimes I think you need the nuances of the doctor because the patient is not completely honest” – resident pediatrics, 4^th^ year*

*“Patients always lie” [laughter of the group]*

This statement, while made humorously, reflected a broader concern among residents about discrepancies between patient-reported information and clinician data. Residents seemed to struggle with these discrepancies and indicated that consultation with other healthcare professionals helped them to gain a better understanding of the situation.

## 4. Discussion and conclusion

This study explored the attitudes and behaviors regarding SDM among residents and medical specialists of seven teaching hospitals in the Netherlands. While most participants supported the idea of SDM, its implementation was hindered by an underlying disease-centered approach, which influenced residents’ and medical specialists’ professional beliefs and decision-making behavior. To our knowledge, this is the first study that identified that disease-centered beliefs not only contribute to residents’ decision-making [17], but influence medical specialists’ decision-making as well. This is relevant information, as it has consequences for future SDM research and implementation.

### Reflection on main findings

Four physicians’ beliefs were identified, each of which appeared to be based on the concept of disease-centeredness. In a disease-centered model, the disease is treated as an entity, separate from the individual. This approach, rooted in Cartesian dualism, has long been dominant in medical education and practice [26,28]. However, growing emphasis on person-centered care highlights the limitations of this paradigm. Disease-centered thinking reinforces physician authority, creating power imbalances in clinical encounters and limiting meaningful patient involvement [27].

The belief that physicians are the primary experts in decision-making can reduce opportunities for patient participation. This belief is reinforced through the current (postgraduate) medical curriculum, which highlights the central role of the medical expert, shaping professional identity and clinical practice [29]. In addition, previous studies have shown that physicians often assume patients lack the necessary health literacy to engage in SDM effectively, a belief that was also reflected in our focus group study, contributing to a power imbalance in patient-doctor encounters [6,18].

Similarly, the perception that disease determines treatment influences treatment planning. Our findings align with previous research showing that MDT meetings primarily focus on clinical data rather than patient preferences [30,31]. This focus on disease can lead to the belief that patient context and preferences are not deemed necessary to make the decision. This is a missed opportunity, because taking patient preferences into account often leads to less overtreatment and contributes to appropriate person-centered care [9,32]. As estimating patients’ preferences often leads to incorrect assumptions, doctors will have to explicitly ask patients about their preferences [32,33].

Participants’ strong belief in a single best treatment for each disease, aligns with a disease-centered approach, which prioritizes biomedical evidence and standardized treatment protocols over individual patient preferences. In this framework, clinical decision-making is guided primarily by objective disease characteristics, with the assumption that an optimal intervention exists for each condition. This perspective may make SDM more challenging, particularly when guidelines strongly favor one approach [34]. This belief can reduce perceived decision-making flexibility, making it less likely that alternative options tailored to patients’ values and preferences are presented by physicians [17].

Physicians’ strong sense of responsibility for achieving the correct diagnosis, selecting the best treatment, and ensuring favorable outcomes can further limit patient involvement in decision-making. When physicians feel legally or ethically accountable for the treatment outcome, they may be reluctant to share decision-making control with patients, particularly in high- risk scenarios [33].

Another factor contributing to this reluctance may lie in how physicians have learned to manage uncertainty. Clinical decision-making involves various forms of uncertainty, and medical training emphasizes reliance on reproducible, measurable data to establish confidence in decisions [35]. This tension was reflected in the remark, *“Patients always lie”*, which seemed to resonate among residents. While the term *‘lie’* is a strong expression, possibly implying distrust, its use may reflect an underlying struggle. Residents, feeling responsible for making the right diagnosis and treatment decisions, may experience uncertainty when confronted with conflicting information. In such cases, they may be more inclined to prioritize clinical data or information from other physicians over patient-reported data, reinforcing a physician-led approach to decision-making [36]. Additionally, uncertainty about treatment outcomes and limited publicized patient-centered outcomes in research can also pressure doctors to continue treatment for longer than is actually necessary or desired [35].

### Strenghts and limitations

A strength of our study is the mixed-method design that allowed us to explore the perceptions and beliefs playing a role in decision-making in detail and nuance. In addition, this is the first study to examine these perceptions and beliefs among both residents and medical specialists, who together shape postgraduate medical education. The main limitation of this study is the low survey reponse rate, which appears to be common among physician surveys [37]. Despite the low response rate, the results of the survey were consistent with findings from other studies and provided a useful basis for the focus group interviews. Another limitation of the survey is the self-reporting, which is susceptible to social desirability bias.

### Conclusion

While SDM is widely endorsed, its implementation in practice is constrained by residents’ and medical specialists’ disease-centered thinking. Addressing this challenge requires a paradigm shift in postgraduate medical education, emphasizing the integration of patient preferences alongside clinical expertise.

### Practice implications

Successful implementation of SDM in postgraduate medical education requires addressing these deeply ingrained disease-centered beliefs. Incorporating training that includes reflection on personal experiences or observed consultations can enhance this awareness [38,39]. Increasing patient involvement in medical training and the development of medical competencies, can further shift the focus from the physician’s role as a medical expert, toward a more person-centered approach [40,41].

Residents in both primary and hospital care have expressed the need for sustained attention to SDM throughout their medical education [42,43]. Since our focus group participants indicated that their ability to apply SDM largely depends on their clinical experience and interactions with supervisors, future research should explore residents’ specific needs regarding SDM and person-centered care training at different stages of their medical education.

## Additional File

The additional file for this article can be found as follows:

10.5334/pme.1465.s1 Supplementary Appendix. Appendix A–Appendix E.
