# Supplementary material for: Barriers to Implementing Shared Decision-Making in Postgraduate Medical Education: The Role of Disease-Centered Beliefs
Source: Perspect Med Educ. 2025 Jul 25;14(1):436–46. doi: 10.5334/pme.1465 (PMC12292059; doi:10.5334/pme.1465)
Supplement: Supplementary Appendix. — Appendix A–Appendix E. [file pme-14-1-1465-s1.pdf]

Supplementary appendix

Appendix A: Results of the survey

Table 1: General characteristics of participants

	Residents *n=127*	Physicians *n=188*
**Age**		
- 20-25	3 (0.5%)	
- 26-30	44 (34.6%)	1 (0.5%)
- 31-35	69 (54.3%)	10 (5.3%)
- 36-40	9 (7.1%)	34 (18.1%)
- 41-45	1 (0.8%)	31 (16.5%)
- 46-50		28 (14.9%)
- 51-55		27 (14.4%)
- 56-60		35 (18.6%)
- 60-65		22 (11.7)
- n.a.	1 (0.8%)	
**Gender**		
- Female	93 (73%)	96 (51,1%)
- Male	34 (27%)	88 (46.8%)
- Other		4 (2.1%)
**Years of residency or work**		
- 1 | 0-5	17 (13.4%)	17 (9.0%)
- 2 | 6-10	17 (13.4%)	32 (17.0%)
- 3 | 11-15	13 (10.2%)	42 (22.3%)
- 4 | 16-20	18 (14.2%)	34 (18.0%)
- 5 | 21-25	19 (14.2%)	38 (20.0%)
- 6 | 26-30	10 (7.9%)	24 (12.8%)
- n.a	33 (26.0%)	1 (0.5%)
**Hospital**		
- General	65 (51.1%)	106 (56.4%)
- Academic	62 (48.8%)	82 (43.6%)
**Specialty**		
- Anesthesiology	1 (0.8%)	5 (2.7%)
- Cardiology	6 (4.7%)	10 (5.3%)
- Cardiothoracic surgery	1 (0.8%)	2 (1.1%)
- Dermatology	-	4 (2.1%)
- Psychology	2 (1.6%)	1 (0.5%)
- General surgery	9 (7.1%)	7 (3.7%)
- General practionar	1 (0.8%)	20 (10.6%)
- Internal medicine	30 (23.6%)	11 (5.9%)
- Intensive Care	3 (2.4%)	6 (3.2%)
- Pediatrics	8 (6.3%)	27 (14.4%)
- Clinical Genetics	2 (1.6%)	6 (3.2%)
- Geriatrics	5 (3.9%)	6 (3.2%)
- E.N.T.	4 (3.1%)	7 (3.7%)
- Pulmonology	10 (7.9%)	6 (3.2%)
- Gastroenterology	1 (0.8%)	2 (1.1%)
- Oral and maxillofacial surgery	-	2 (1.1%)
- Neurosurgery	1 (0.8%)	-
- Neurology	6 (4.7%)	10 (5.3%)
- Obstetrics and gynaecology	7 (5.5%)	18 (9.6%)
- Ophtalmology	3 (2.4%)	-
- Orthodontics	2 (1.6%)	-
- Orthopedic surgery	4 (3.1%)	-
- Gerontology	-	2 (1.1%)
- Plastic surgery	1 (0.8%)	-
- Psychiatry	1 (0.8%)	9 (4.8%)
- Radiology	1 (0.8%)	2 (1.1%)
- Radiotherapy	1 (0.8%)	8 (4.3%)
- Rheumatology	4 (3.1%)	5 (2.7%)
- Rehabilitation medicine	1 (0.8%)	2 (1.1%)
- Emergency medicine	10 (7.9%)	5 (2.7%)

- Urology	1 (0.8%)	5 (2.7%)

Table 2: Current practice of SDM

	Resident (n=127)	Physician (n=188)
**When making a decision about a patient's diagnosis or treatment, which of the following best describes how you typically make such a decision?**		
- The doctor decides	0 (0%)	1 (0.5%)
- The doctor decides but takes the patient's opinion into account	45 (35.4%)	20 (10.6%)
- The patient and doctor make a joint decision	46 (36.2%)	116 (61.7%)
- The patient decides but takes the doctors opinion into account	32 (25.2%)	40 (21.3%)
- The patient decides	4 (3.1%)	11 (5.8%)
**I explain the advantages of the treatment options**		
- Never	0 (0%)	1 (0.5%)
- Rarely	0 (0%)	1 (0.5%)
- Sometimes	1 (0.8%)	1 (0.5%)
- Regularly	13 (10.2%)	14 (7.4%)
- Generally	44 (34.6%)	58 (30.9%)
- Always	69 (54.3%)	113 (60.1%)
**I explain the disadvantages of the treatment options**		
- Never	0 (0%)	1 (0.5%)
- Rarely	1 (0.8%)	0 (0%)
- Sometimes	1 (0.8%)	7 (3.7%)
- Regularly	19 (15.0%)	11 (5.9%)
- Generally	51 (40.2%)	63 (33.5%)
- Always	55 (43.3%)	106 (56.4%)
**I explain the advantages and disadvantages of the treatment options equally**		
- Never	0 (0%)	2 (1.1%)
- Rarely	3 (2.4%)	0 (0%)
- Sometimes	17 (13.4%)	18 (9.6%)
- Regularly	33 (26.0%)	37 (19.7%)
- Generally	55 (43.3%)	63 (33.5%)
- Always	19 (15.0%)	68 (36.2%)
**I check whether the patient understands the advantages of the treatment options**		
- Never	1 (0.8%)	1 (0.5%)
- Rarely	4 (3.1%)	6 (3.2%)
- Sometimes	21 (1.5%)	19 (101%)
- Regularly	31 (24.4%)	32 (17.0%)
- Generally	52 (40.9%)	70 (37.2%)
- Always	18 (14.2%)	60 (31.9%)
**I check whether the patient understands the disadvantages of the treatment options**		
- Never	1 (0.8%)	1 (0.5%)
- Rarely	4 (3.1%)	5 (2.7%)
- Sometimes	19 (15.0%)	20 (10.6%)
- Regularly	32 (25.2%)	34 (18.1%)
- Generally	52 (40.9%)	65 (34.6%)
- Always	19 (15.0%)	63 (33.5%)
**I explain the differences between the treatment options**		
- Never	0 (0%)	1 (0.5%)
- Rarely	1 (0.8%)	2 (1.1%)
- Sometimes	8 (6.2%)	12 (6.4%)
- Regularly	28 (22.0%)	24 (12.8%)
- Generally	59 (46.5%)	82 (43.6%)
- Always	31 (24.4%)	67 (35.6%)
**The patient is given the opportunity to ask questions about the treatment options**		
- Never	0 (0%)	1 (0.5%)
- Rarely	0 (0%)	0 (0%)
- Sometimes	1 (0.8%)	1 (0.5%)
- Regularly	7 (5.5%)	7 (2.7%)
- Generally	27 (21.3%)	27 (19.7%)
- Always	92 (72.4%)	142 (75.5%)
**At the beginning of the conversation I say that there is a choice regarding treatment**		
- Never	4 (3.1%)	2 (1.1%)
- Rarely	13 (10.2%)	13 (6.9%)
- Sometimes	27 (21.3%)	23 (12.2%)
- Regularly	29 (22.8%)	31 (16.5%)
- Generally	31 (24.4%)	53 (28.2%)
- Always	23 (18.1%)	66 (35.1%)

**I say that it matters what the patient considers important**		
- Never	2 (1.6%)	1 (0.5%)
- Rarely	9 (7.1%)	5 (2.7%)
- Sometimes	15 (11.8%)	15 (8.0%)
- Regularly	37 (29.1%)	25 (13.3%)
- Generally	41 (23.3%)	63 (33.5%)
- Always	23 (18.1%)	79 (42.0%)
**I check whether I understood correctly what is important to the patient**		
- Never	2 (1.6%)	2 (1.1%)
- Rarely	8 (6.3%)	6 (3.2%)
- Sometimes	23 (18.1%)	18 (9.6%)
- Regularly	41 (23.3%)	34 (18.1%)
- Generally	32 (25.2%)	74 (39.4%)
- Always	21 (16.5%)	54 (28.7%)
**I help the patient to weigh the advantages and disadvantages of the treatment options**		
- Never	1 (0.8%)	2 (1.1%)
- Rarely	3 (2.4%)	1 (0.5%)
- Sometimes	15 (11.8%)	9 (4.9%)
- Regularly	44 (34.6%)	30 (16.0%)
- Generally	49 (38.6%)	83 (44.1%)
- Always	15 (11.8%)	63 (33.5%)
**I give the patient time to weigh the advantages and disadvantages of the treatment options (during or after the conversation)**		
- Never	0 (0%)	1 (0.5%)
- Rarely	8 (6.3%)	2 (1.1%)
- Sometimes	15 (11.8%)	12 (6.4%)
- Regularly	55 (34.6%)	38 (20.2%)
- Generally	36 (28.3%)	71 (37.8%)
- Always	24 (18.9%)	64 (34.0%)
**I ask the patient what is important to him/her**		
- Never	1 (0.8%)	0 (0%)
- Rarely	6 (4.7%)	7 (3.7%)
- Sometimes	24 (18.9%)	13 (6.9%)
- Regularly	44 (34.6%)	32 (17.0%)
- Generally	35 (27.6%)	76 (40.4%)
- Always	17 (13.4%)	60 (31.9%)

Table 3: SDM in medical education

	Resident n=127	Physician n=188
**I (get) supervise(d) on shared decision making**		
- Yes	24 (18.9%)	83 (44.1%)
- No	50 (39.4%)	18 (9.6%)
- Sometimes	53 (41.7%)	87 (46.3%)
**During patient consultations, several treatment options are discussed**		
- Yes	75 (59.0%)	161 (85.6%)
- No	5 (3.9%)	3 (1.6%)
- Sometimes	47 (37.0%)	24 (12.8%)
**I (get) encourage(d) to practice SDM**		
- Yes	37 (29.1%)	123 (65.4%)
- No	19 (15.0%)	9 (4.8%)
- Sometimes	71 (55.9%)	56 (29.8%)
**My supervisors usually practice SDM**		
- Yes	48 (37.8%)	
- No	11 (8.7%)	
- Sometimes	68 (53.5%)	
**In our department SDM is common practice**		
- Yes	79 (62.2%)	135 (71.8%)
- No	48 (37.8%)	53 (28.1%)
**Within our department there are several role models with regard to SDM**		
- Yes	62 (48.8%)	89 (47.3%)
- No	65 (51.2%)	99 (52.7%)
**SDM should be a recurring (mandatory) part of medical education**		
- Yes	104 (81.9%)	176 (93.6%)
- No	23 (18.1%)	12 (6.4%)
**I did a training / course regarding SDM**		
- Yes	7 (5.5%)	33 (17.6%)
- No	120 (94.4%)	155 (82.4%)
**A training / course regarding SDM is a integral part of my medical education**		
- Yes	12 (9.4%)	22 (11.7%)

- No	115 (90.6%)	166 (88.3%)

Table 4: Attitudes regarding SDM

	Resident N=127	Physician N=188
**Most patients do not want to make shared decisions, they want the doctor to make the decision**		
- True	60 (47.2%)	51 (27.1%)
- False	55 (43.3%)	116 (61.7%)
- Not sure	12 (9.4%)	21 (11.2%)
**SDM is better for the patient**		
- True	107 (84.3%)	155 (82.4%)
- False	8 (6.3%)	11 (5.9%)
- Not sure	12 (9.4%)	22 (11.7%)
**SDM is better for the doctor**		
- True	89 (70.1%)	121 (64.4%)
- False	17 (13.4%)	31 (16.5%)
- Not sure	21 (16.5%)	36 (19.1%)
**SDM takes more time**		
- True	92 (72.4%)	116 (61.7%)
- False	28 (22%)	59 (31.4%)
- Not sure	7 (5.5%)	13 (6.9%)
**SDM makes a consultation unnecessarily complicated**		
- True	9 (7.1%)	6 (3.2%)
- False	103 (81.1%)	166 (88.3%)
- Not sure	15 (11.8%)	16 (8.5%)
**I prefer SDM if possible**		
- True	118 (92.9%)	178 (94.7%)
- False	7 (5.5%)	3 (1.6%)
- Not sure	2 (1.6%)	7 (3.7%)
**I use decision aids**		
- True	38 (29.9%)	67 (35.6%)
- False	77 (60.6%)	105 (55.9%)
- Not sure	12 (9.4%)	16 (8.5%)
**I am familiar with the national SDM campaign**		
- True	47 (37.0%)	115 (61.2%)
- False	76 (59.8%)	62 (33.0%)
- Not sure	4 (3.1%)	11 (5.9%)
**I am competent in SDM**		
- True	85 (66.9%)	158 (84.0%)
- False	24 (18.9%)	10 (5.3%)
- Not sure	18 (14.2%)	20 (10.6%)
**I need more skills regarding SDM**		
- True	66 (52.0%)	52 (27.7%)
- False	44 (34.6%)	107 (56.9%)
- Not sure	17 (13.4%)	29 (15.4%)
**I need more substansive training about SDM**		
- True	53 (41.7%)	48 (25.5%)
- False	59 (46.5%)	109 (58.0%)
- Not sure	15 (11.8%)	31 (16.5%)
**I need (more) feedback from my supervisors on SDM**		
- True	61 (48%)	
- False	53 (41.7%)	
- Not sure	13 (10.2%)	
**I am willing to invest time to enhance my knowledge and skills in SDM**		
- True	95 (74.8%)	129 (68.6%)
- False	19 (15.0%)	26 (13.8%)
- Not sure	13 (10.2%)	33 (17.6%)

Appendix B: Main research questions focus group interviews

1. Is there a ‘best’ treatment? What determines if a treatment option is considered best?
2. What is the role of the patient in shared decision making?
3. What is the role of other health care professionals in shared decision making?
4. What do you need to succesfully implement shared decision making in medical education?

Appendix C: COREQ checklist:

**Domain 1**	**Research team and reflexivity**	
1. Interviewer	GW (author)	LW (author)
2. Credentials	PhD	MD
3. Occupation	Policy and implementation advisor	Resident internal medicine<br>fellow hematology
4. Gender	Female	Female
5. Experience and training	Conduncting FGI's	Medical and substansive knowledge
6. Relationship participants	None	Some of the participants were well-known colleagues
7. Participant knowledge of the interviewer	Name, age, occupation	Name, age, occupation
8. Interviewer characteristics	Reasons and interests in the research topic	Reasons and interests in the research topic
**Domain 2**	**Study design**	
9. Methodological orientation and theory	Thematic analysis	
10. Sampling	Convenience	
11. Method of approach	Email	
12. Sample size	Survey: 315 participants, FGI's: 21 participants	
13. Non-participation	16 participants could not complete the survey because they did not have live contact with patients.<br>3 participants could not join the focus group interviews due to workload.	
14. Setting of data collection	Data from the survey was collected online and stored in an excelfile.<br>Data from the focus group interviews were collected in a meeting room in the hospital and recorded online via teams. Data was stored in Teams.	
15. Presence of non-participants	No	
16. Description of sample	The following sample criteria were collected at baseline: age (in clusters of 5 years), gender, year of education or workexperience, type of hospital (academic or general hospital)	
17. Interview guide	There were 4 main research questions prepared prior to the FGI's, which were altered if necessary and used to structure the subsequent FGI's.<br>It was not pilot tested.	
18. Repeat interviews	No	
19. Audio/visual recording	Both audio and visual recording via Teams and via Ipad.	
20. Field notes	Yes by the author who attended the FGI's and by an author who viewed the recorded FGI's.	
21. Duration	Excel, Microsoft Word.	
22. Data saturation	Yes	
23. Transcripts returned	No	
**Domain 3**	**Analysis and findings**	
24. Number of data coders	2	
25. Description of the coding tree	Yes, in appendix B.	
26. Derivation of themes	4 main themes were identified in advance, based on a previous study. These themes were altered after testing it on the transcripts. The final themes were derived from the data of the FGI's.	
27. Software	Excel, Microsoft Word	
28. Participant checking	Not yet. Participants will be sent a copy of the main results by e-mail.	
29. Quotations	Yes	

presented	
30. Data and findings consistent	Yes
31. Clarity of major themes	Yes
32. Clarity of minor themes	Yes

Appendix D: Thematic Analysis

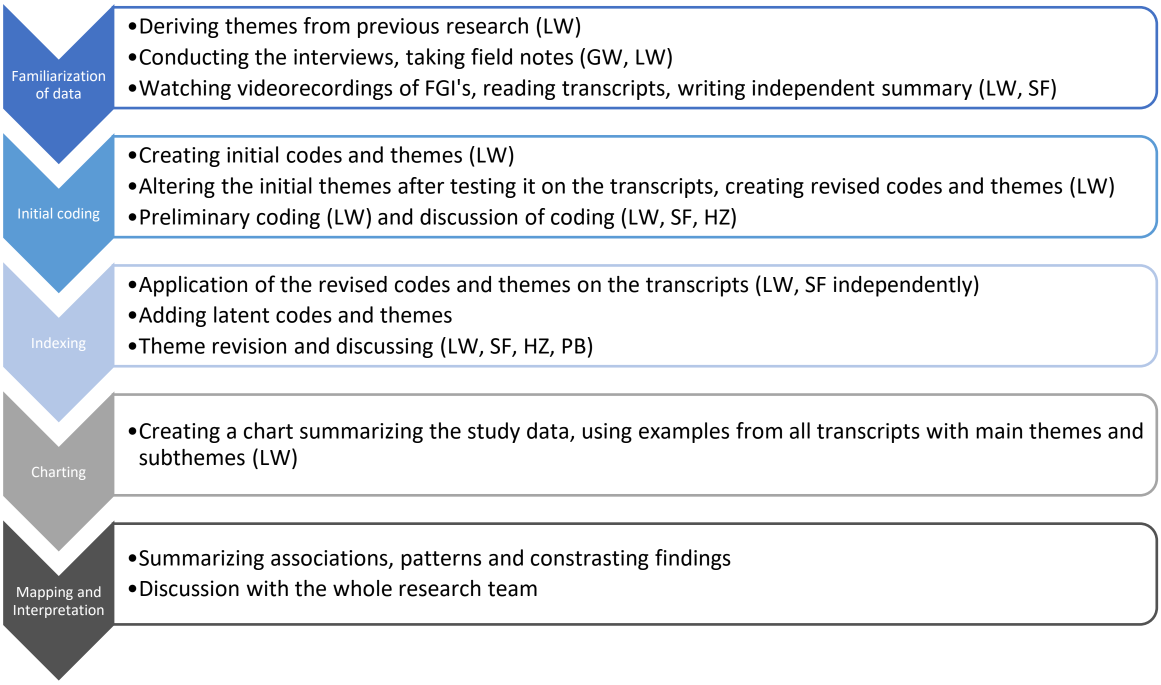

GW = Author
LW = Author
SF = Author
PB = Author
HZ = Hester van der Zaag

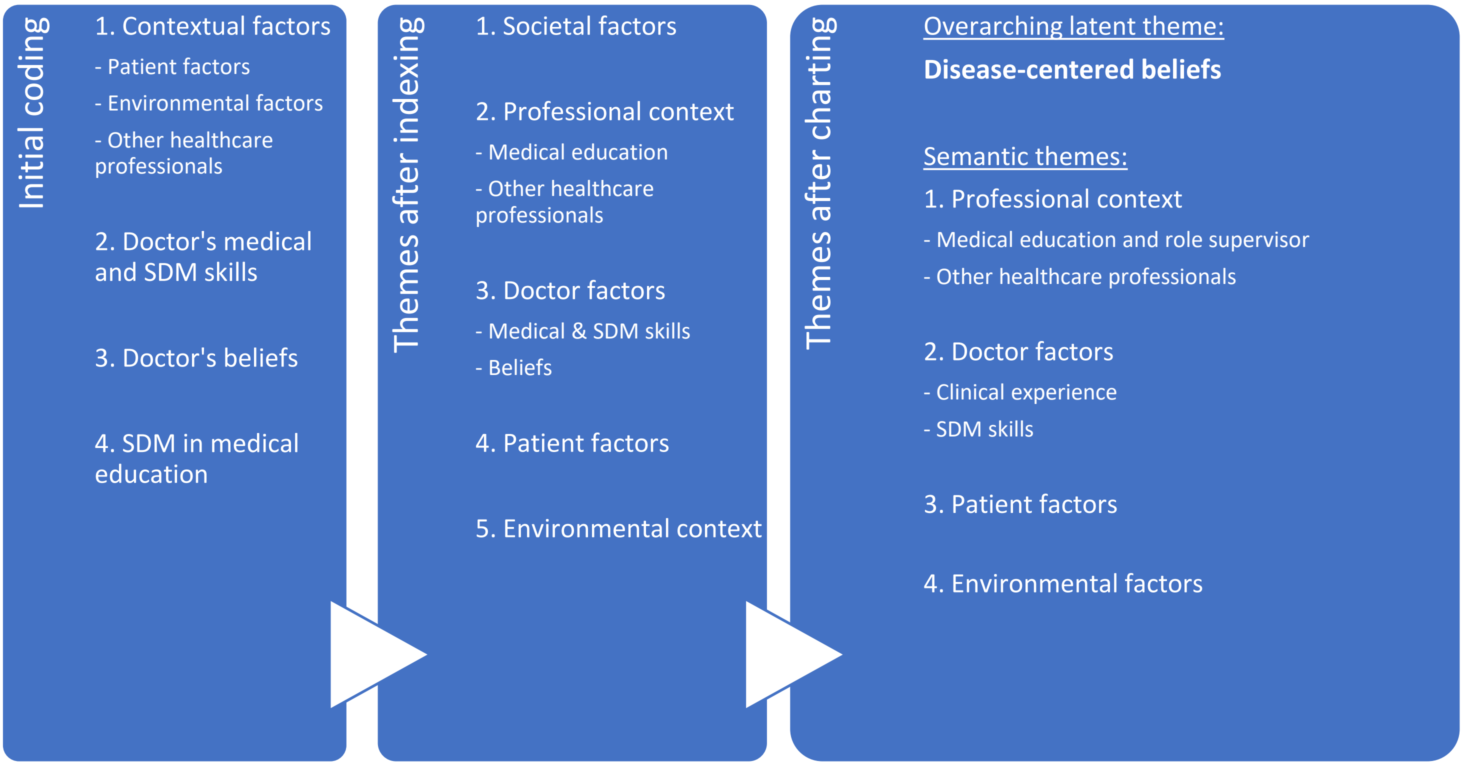

Appendix E: Themes and quotes of participants

Semantic themes and quotes	Latent codes and themes
**Theme 1: Professional context**<br>1.1 Medical education and role supervisor<br>*Q1 "If you are going to consult with a supervisor to discuss the options, the supervisor usually makes the decision. Then you have to sell that to the patient and dress it up in such a way that it seems you made the decision together" – resident orthopedic surgery, 4th year*<br>*Q2 "I have certain supervisors who make the decision for me, while I prefer to have several options and discuss this with the patient" – resident rheumatology, 4th year*<br>*Q3 "As a relatively young doctor I tend to stick more to the standard protocols so to speak, as in my opinion, that's when I provide the best care. But to broaden that perspective.. well you need the nuance of your supervisor for that" – resident rheumatology, 4th year*<br>*Q4 "More and more people are paying attention to SDM, so I think it will be easier for us to incorporate this into medical education" – resident E.N.T., 1st year*<br>*Q5 "I think most of you have heard [name] talk about this [SDM], and he speaks of it so well and gives such great examples that you are quickly tempted to try it too, just because he has such a nice story about it and has such positive experience with it. I think it is less motivating, seeing rigid old specialists doing it very differently. So a good example is very useful" - resident orthopedic surgery, 4th year.*<br><br>1.2 Role of other health care professionals<br>*Q6 "Because there is a different hierarchy… they tell different things to the rheumatology nurse than to me, and that uhm sometimes really helps to make different choices" – rheumatologist, 26-30 years in profession*<br>*Q7 "We have an oncology nurse, who is a real support…she often takes patients to her own room after the conversation and that is*	**Main overarching theme: disease-centeredness**<br><br>**Belief 1: The doctor is the expert**<br>*Q24 "Before the patient can make an informed choice.. well, there is a reason why doctors have such a long education before they are able to make a choice like that." – resident E.N.T., 1st year*<br>*Q25 "I think in practice the story is presented in such a way that you push the patient in the way you want to go, so yes, I think in practice the feeling of making decisions together is applied more than actually making decisions together" – resident orthopedic surgery, 4th year.*<br>*Q26 "Well we see ourselves as experts in that, so to speak, and I think that could be a barrier for shared decision making. You really give the patient a voice, uhm, while you actually think: well the patient is not necessarily the one who knows most about it, right?" – ER specialist, 16-20 years in profession*<br>*Q27 "I think many patients assume that it is a strong advice, and not a choice" - resident E.N.T., 1st year*

very helpful, because sometimes new aspects come to light that patients are concerned about, to which we can respond" – orthopedic surgeon, 11-15 years in profession<br>*Q8 "You [as GP] often know the patient better or, well, how someone actually feels about life, if someone has difficulty expressing himself… so the role of the GP is important" – GP, 31-35 years in profession*	
**Theme 2: Doctor factors**<br>2.1 Clinical experience<br>*Q9 "It is difficult to find out what is important for the patient to take into account, when you have little experience with the treatment" – resident rheumatology, 4th year*<br>*Q10 "You also have to be very aware of the pros and cons of the treatments and I don't think we always know that exactly" – ER specialist, 21-25 years in profession*<br>*Q11 "I think you approach a conversation very differently if you have medical knowledge and experience. If you don't have experience yet, you may have the impression that you just have to do what your supervisor says" – resident geriatrics, 2nd year*<br>*Q12 "Don't you think it [SDM] is also about experience? Look, as a resident you have to act on behalf of your supervisor - orthopedic surgeon, 11-15 years in profession*<br>*Q13 " I think it is more difficult for residents, they do short-term internships in different departments… and are confronted with a new type of patients with new protocols and treatments etc, before they have noticed how those treatments work, they have to start another internship.. You are the advisor but you never evaluate the outcome of your own advise" - gynaecologist, 30-35 years in profession*<br><br>2.2 SDM skills<br>*Q14 "I think the pitfall for us is often that we outline the pros and cons and tell the patient to make the decision, but that is different from really asking 'but what is important to you?'" – rheumatologist, 26-30 years in profession*<br>*Q15 "And uhm, it turns out that you think you are doing it [SDM], but that you are actually doing very little or just one step and nothing more. So that was confrontational" – rheumatologist, 26-30 years in training*<br>*Q16 "I think people often have the idea that they do it, but I think few people actually start the conversation with 'There will be a decision moment'– resident E.N.T., 1st year*	**Belief 2: The disease determines the treatment**<br>*Q28 "We are of course trained in a certain way, uhm, that we had to give a treatment proposal for a certain medical condition" – orthopedic surgeon, 11-15 years in profession*<br>*Q29 "I notice in MDT's, that it is already being decided what will be done.. and only then is it discussed with the patient.. while actually the entire treatment plan is already esthablished." – resident geriatrics, 2nd year*<br>Q30 *"To return to those MDT's where the treatment plan has already been completely determined. I don't think that is surprising. Very difficult cases are discussed and then you will go sit together with a group of specialists who, for example, all treat the same disease" – resident E.N.T., 4th year*<br>*Q31 "Actually, a consultation always has a treatment plan, right" – resident orthopedic surgery, 4th year*<br>*"Yes, but the question then is whether the process of SDM.. whether the patient preferences are always discussed. The treatment plan wìll be made, but.. – resident pediatrics, 4th year. [several residents laugh]*<br>*Q32 "Usually we speak most of the time: 'you can do this, you can do that, these are the options'. But that the patient talks much about his own situation? We only ask for the information we need: 'How fit is someone?', 'Can he handle this operation or this chemotherapy?'. But the patient does not share a lot in the conversation." – resident internal medicine, 1st year*
**Theme 3: Patient factors**<br>*Q17 "Intelligence level is also such a complicated one, isn't it? [agreement in the group]… So you quickly ask too much of the patient…you explain things, but very little is recalled" – psychiatrist, 11-15 years in profession*<br>*Q18 "Sometimes there are people who say, well, I don't want a certain chemotherapy…this is then discussed during rounds or in the MTM and the patient specific opinion is introduced, but that's only because the patient is so upfront about it" – resident geriatrics, 2nd year.*<br>*Q19 "You discuss the treatment options with the patient, first of all IF you can discuss it with the patient, because you have to act quickly otherwise the patient will suffer" – ER specialist, 16-20 years in profession*<br>*Q20 "Sometimes there is a lot of information to give to a patient and you have to phase it, otherwise it becomes far too much… well because of the amount of information, but also because it is very emotionally confrontational" – gynaecologist, 31-35 years in profession*	**Belief 3: There is one best treatment for each disease**<br>*Q33 "It depends if the treatment options are equal. If it is a choice like: 'well we have option A and we have option B but we don't necessarily know which is better, what is your preference?'. Ehm but if I know option A is better, well, am I going to give the patient a choice then?" – resident pediatrics, 4th year*<br>*Q34 "As a relatively young doctor I just want to act according to the protocol, as in my opinion, that's when I provide the best care" – resident rheumatology, 4th year*<br>*Q35 "Often you already know: this is the best choice for this problem for the patient, uhm, so which choice is there? I don't think with every treatment there is, uhm well, so much to choose" – ER specialist, 16-20 years in profession*<br>*Q36: "If we see a new patient with rheumatoid arthritis… you have a lot of different remedies that you could choose from, but in our treatment protocol it is methotrexate. That is simply the first choice and there are quite a few reasons why that is the case, but you may wonder how much choice there actually is for the patient". – rheumatologist, 26-30 years in profession*
**Theme 4: Environmental context**<br>*Q21 "You can hardly explain and discuss everything with the patient in that amount of time, in such a way that it is actually understood and perhaps even discussed at home… you cannot actually ask a*	**Belief 4: The doctor is responsible**<br>*Q37 "I think this happens less with younger patients, especially in oncology. Yes you always have a choice of course, you always have to decide together of course, but ehm, doing nothing sometimes*

patient to make a decision in 5 or 10 minutes" – resident E.N.T., 1st year<br>*Q22 "I think SDM is easier in the GP's office, more common I think. In the hospital, the approach is sometimes different. People are already here… the decision is do you still want to go to the hospital?" – GP in training, 2nd year.*<br>*Q23 "Decision aids on paper, uhm diagrams, uhm if you are talking about numbers needed to treat, for example, these are concepts that we sometimes find difficult as doctors, let alone patients… so we need clear numeric information about pros and cons" – ER specialist, 16-20 years in profession*	*means that the patient dies" – resident general surgery, 5th year*<br>*Q38: "What someone wants to achieve, what is important to someone, that is frequently discussed with us, I think, because the treatment is not urgent and you have to defend yourself if it goes wrong and you did it anyway" – resident orthopedic surgery, 4th year*<br>*Q39: "When you have had a major revision because you have an infection and after that major surgery you get something minor, then it is a terrible shame to give up, also for the patient… In the beginning we frequently ask: do you want all this?, but at a certain moment, yeah, I think most of us do not have that conversation again. [confirmatory sounds]" - resident orthopedic surgery, 4th year.*<br>*Q40: "Then I finally called the GP and it was a completely different story than what the patient had told me. So sometimes I think, you need the nuances of the doctor because the patient is not completely honest" – resident pediatrics, 4th year "Patients always lie" [laughter of the group].*
